# The use of a microprocessor for flexible automation of an experimental procedure

**DOI:** 10.1155/S1463924685000165

**Published:** 1985

**Authors:** M. S. Sheya, C. Riley

**Affiliations:** 1 Department of Electrical Engineering University of Dar es Salaam PO Box 35131 Dares Salaam Tanzania; 2 Centre for Medical Research The University of Sussex Mantell Building Falmer, Sussex Brighton BN1 9RF UK


Journal of Automatic Chemistry/*Journal of Clinical Laboratory Automation*, Vol. 7, No. 2 (Apr–Jun 1985), pp. 69–73

### The use of a microprocessor for flexible automation of an experimental procedure

**M. S. Sheya**
*Department of Electrical Engineering, University of Dar es Salaam, PO Box 35131, Dar es Salaam, Tanzania*

**and C. Riley**
*Centre for Medical Research, The University of Sussex, Mantell Building, Falmer, Brighton, Sussex BN1 9RF, UK*

### Introduction

During the development phase of a new design of multichannel blood-analysis machine, it was necessary to perform empirical tests to determine exact volumes of specimen to be sampled and volumes of reagents and diluent to be dispensed during the normal run of the machine. This is because sample volumes measured in clinical laboratory situations are often in the range of microlitres, and great precision and care is needed in handling such small amounts. From the sampling and dispensing mechanisms of the system, fluid was led through PVC tubing to a multichannel peristaltic pump (the pump's outlet was connected to an input of a z-path cuvette), and finally to drain [1]. The pump was driven by a stepper motor. This is an electromechanical device used here because of the good properties inherent in its stepwise motions when used for precise positioning of objects [2–4]. Thus the pump-stepper motor assembly aided sampling, mixing of reactants, cleansing and draining of the reaction channel during each process cycle.

To ensure a high degree of precision it was imperative to establish the most suitable working parameters of the pump-stepper motor assembly. Hence it became necessary to run the pump at variable speeds and number of rotations until the appropriate volumes were obtained. At first, this task was accomplished manually and proved to be rather tedious and also unreliable. Accordingly to perform a desired preprogrammed sequence of operations, an electromechanical cam-timer was introduced. This served to switch 'on' and 'off' a train of square-wave pulses from a function generator at predetermined intervals. This method, although straightforward, was of course rather inflexible.

To overcome the disadvantages inherent in the above techniques, microprocessor control was introduced, an Intel 8080 microprocessor was used to run the pump-stepper motor assembly in the STEP, REV and PRE-PROGRAMMED modes by issuing simple keyboard commands. For ease of program design, subhigh-level language PL8080 was used [5]; this language has all the features of structured programming. Program development was done on time-sharing basis, with the aid of an ICL 1902s mainframe computer which has a cross compiler. The compiled object program in Intel 8080 object code was then down-loaded into the microcomputer kit for debugging, testing and running.

### Experimental set-up

As a prerequisite to using the pump-stepper motor assembly, a suitable means of control was needed. It was also necessary to provide an appropriate d.c. power-source and a motor-drive board, including compensating and suppression networks. Figure 1 is a block diagram of the experimental set-up. In its normal mode of operation, the stepper motor will take a single step in response to each command pulse at the input of its drive circuitry. The step angle taken will vary according to the design of the motor. The microprocessor therefore issues a train of TTL-compatible pulses to the motor-drive board; the latter translates the command pulses into an alternate switching sequence of motor windings, thus creating discrete motions of the motor. The time taken to accomplish a requested task depends mainly upon the frequency of the input pulses and the step angle taken by the motor in response to each command pulse. The motor we chose was an Impex stepper motor, which has a full angle deflection of 1·8° per step [4]. Diluent, reagent and patient sample were propelled by a multi-channel peristaltic pump to the z-path cuvette of a colorimeter where observation of the coloured reaction product took place. Following a predetermined observation time, the reaction mixture was then pumped out to drain from the cuvette. Other tasks performed by the microprocessor included control of sample sequencing activities and probe transfer operations.

### Stepper motor driver

The pump-stepper motor assembly was controlled by the DRIVER program which had four main components: these are macros, utility routines, command-recognizing routines and command-handling routines (figure 2). On entry, the DRIVER initializes system variables, I/O ports, default motor parameters then prompts the user by printing the Header on the screen. The DRIVER then enters an endless loop, repeatedly scanning the keyboard. In its normal operation the DRIVER accepts and responds to keyboard commands and interacts with the user through a console device. Only user commands defined in the DRIVER command repertoire are accepted. For each valid command the DRIVER responds either by displaying a message or an action being taken. A built-in HELP command displays the available modes of operation and the existing commands. Commands can be issued either on-line (i.e. while the motor is running) or off-line (i.e. when the motor is at stand-still). These commands are mutually exclusive. When writing the DRIVER, structured and modular programming was used. A linear search matched commands and a case

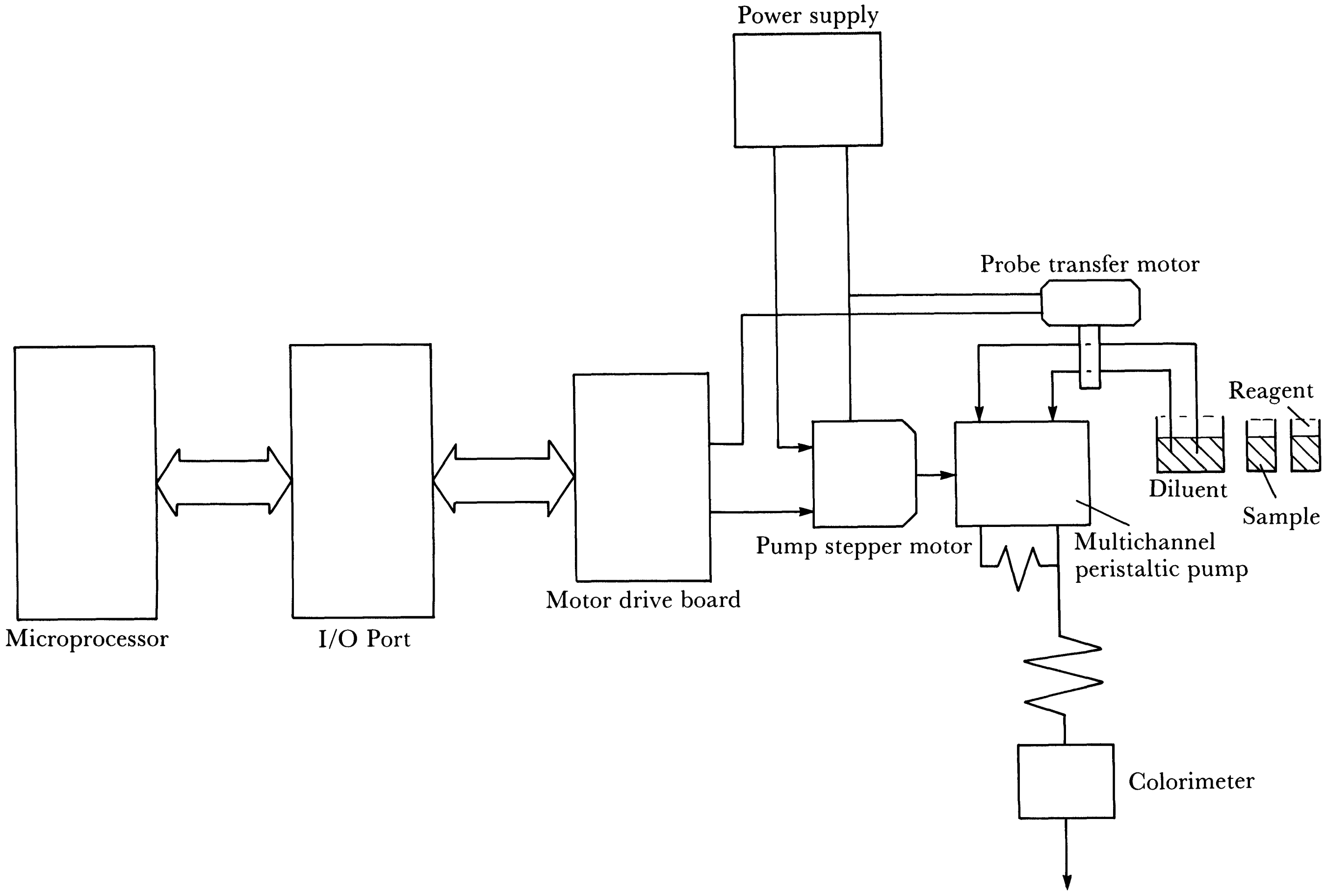

*Figure 1. Experimental arrangement*

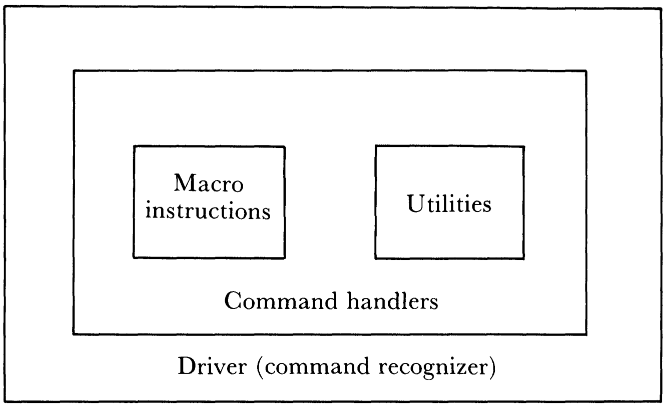

*Figure 2. Hierarchical structure of motor driver.*

statement was used to invoke command-handling routines. For ease of readability, the DRIVER and its main components are described in pseudo-Pascal algorithmic language. Figures 3 and 4 give the main body of the program and a branch-table of command-handling routines. For monitoring the input/output functions of the DRIVER, four distinct message types are available viz. informative, instructive, warning and error messages. The informative messages are automatically invoked upon first entry or upon mode selection. These messages consist mainly of Headers. Instructive messages are invoked by typing appropriate keyboard commands and are used to the user unfamiliar with the DRIVER mode of operations. Warning messages are invoked if the user tries to override the limits of predefined input parameters. Error messages, on the other hand, are displayed if the set conditions have actually been violated and in this case the issued command is automatically ignored.

### Off-line command handlers

These are grouped in auxiliary and main subroutines. Auxiliary subroutines perform simple tasks of changing logic states: for example to reverse motor direction or to change stepping mode and to display information. These subroutines are of a general class of SET/RESET and of a MESSAGE handler. A SET subroutine changes an output state from logic '0' to logic '1', leaving the rest of the output byte unchanged. A RESET subroutine is the opposite of a SET subroutine. The three modes of operations are defined by the main subroutines as described below.

(1) STEP mode is entered by invoking STEPFUNCTION (figure 5). This subroutine runs the motor in a uni-step or multi-step manner. On entry the routine expects the user to specify the number of steps the motor should rotate. Only a valid step count (1 to 255) will be accepted. The motor used had a step angle of 1·8° and hence requried 200 steps/rev. STEPFUNCTION instigates an error message if input conditions are violated.

```
program driver (input, output);
begin
  initialize;
  run:=TRUE;
  while run do
    begin
      scan-keyboard;
        if seen then
          begin
            count:=0;
            while count<maxcount do
              begin
                if commandtable(count)=inputcommand then
                  begin
                    invoke-associated-command-implementer;
                    count:=maxcount
                  end else
                  count:=count+1
              end
          end
    end:
    begin
      writeln('end of session')
    end
end.
```

*Figure 3. Main body of the DRIVER program.*

```
procedure commandhandler;
  begin
    case count of
      0: stepfunction;
      1: revfunction;
      2: preprogfunction;
      3: delaycount;
      4: fullstep;
      5: halfstep;
      6: forward;
      7: reverse;
      8: help;
      9: abandone
    end
  end
```

*Figure 4. Branch-table of command-handling routines.*

```
procedure stepfunction;
  begin
    get-count;
      if trap:=GO then
        nextoperation:=TRUE;
        while nextoperation do
          begin
            repeat
              stepmotor;
                detect-external-event;
                stepcount:=stepcount-1;
            until stepcount=EXHAUSTED;
            operationcount:=operationcount-1;
            if operationcount:=EXHAUSTED then
              nextoperation:=FALSE else
              stepcount:=inputcount
          end
  end
```

*Figure 5. Uni-step/multi-step routine.*

(2) REV mode is entered by invoking REVFUNCTION (figure 6). The number of revolutions the motor is to rotate is specified by the user. As a valid input count is any number between 1 and 255. The basic step count/rev is a function of the motor in use and this is stored in the microcomputer's memory. REVFUNCTION instigates an error message if input conditions are violated.

(3) PREPROGRAMMED mode is invoked by PREPROGFUNCTION and performs a desired sequence of operations, such as driving a small synchronous motor coupled to a kinematic linkage to more sample and reagent probes, as well as control diluent dispensing (figure 7). The sequence is completed by performing the necessary cleaning task, thus making the machine ready for the next sequence of operations.

```
procedure revfunction;
  begin
    get-count:
    if trap=GO then
      nextoperation:=TRUE;
      while nextoperation do
        begin
          repeat
            repeat
              stepmotor;
              detect-external-event;
              stepcount:=stepcount-1
            until stepcount=EXHAUSTED;
            stepcount:=stepsperrev;
            revcount:=revcount-1
          until revcount=EXHAUSTED;
          operationcount:=operationcount-1;
          if operationcount=EXHAUSTED then
            nextoperation:=FALSE else
            revcount:=inputcount
        end
  end
```

*Figure 6. Uni-rev/multi-rev routine.*

This routine also accepts input parameters, for example the desired motor speed and the number of revolutions. In all the above three cases, while the motor is running, the keyboard is scanned for possible detection of an on-line command and the end of an operation is signalled by a bell prompt.

### On-line command handlers

These are invoked while the motor is running. The available on-line commands are REPEAT (to repeat a task), SLOWSPEED (to decelerate the motor), FAST-SPEED (to accelerate the motor) and ABANDON (to terminate a task). DETECT (figure 8) is the on-line command-recognizing routine and is invoked in all three modes of operations. On entry, DETECT saves CPU status then scans through the keyboard for a valid command; if successful, an appropriate action is taken on-line. To reduce access time, macros were used in place of subroutine calls. At the end of execution, DETECT restores the CPU status.

```
procedure preprogfunction;
  begin
    synchromotor;
      (*move probe to reagent and sample containers*)
    stepcount:=stepsperrev;
    revcount:=count1;
    rotatepump;
      (*slowly to dispense reagent and patient sample*)
    synchromotor;
      (*return probe into distilled water trough*)
    transfermixture;
      (*rotate pump fast to dispense diluent and reaction mixture to cuvette*)
    delay;
      (*fifteen seconds to observe reaction rate*)
    stepcount:=stepsperrv;
    revcount:=count2;
    rotatepump
      (*fast to rinse the system*)
end
```

*Figure 7. Preprogrammed sequence of operations.*

### Conclusions

In order to establish motor-pump working parameters necessary for dispensing correct volumes of reaction mixtures, an experimental set-up was built. As an efficient and flexible means of arriving at the desired volumes, software control was used. With the aid of an Intel 8080 microprocessor, faster and precise results were obtained and the task, that would otherwise be arduous, was greatly simplified. The application program was general-purpose and could run the motor in any desired number of steps or revolutions and at variable speeds. The program was also designed to handle a pre-programmed sequence of operations and for on-line command detection and execution.

### Summary

The use of the microprocessor controlled, stepper-motor driven peristaltic pump described above permitted the development of a simplified flow-injection system suitable for use in clinical chemistry. Aspiration of sample by the pump proved to be remarkably precise; for example, it was possible to measure serum albumen using bromocresol green on samples as small as 250 nl with a coefficient of variation of 2%.

Such precise volumetric control of liquids by a simple and inexpensive device suggests a wide range of uses in metering reagents both in analytical chemistry and in process control. It is surprising that similar devices are not already widely used.

### References

1. Sheya, M. S., Microprocessor control of discrete clinical chemical analysers (D.Phil. thesis, University of Sussex, 1980).
2. Freeman, M., *Electrical Equipment*, **18** (1979).
3. Editorial (1978), *OEM Design* (April).
4. *Impex Stepper Motors Catalogue* (Impex Electrical, Market Road, Richmond, Surrey, UK, 1972).
5. Foster, J. M., *Programming Language PL8080* (Royal Signals and Radar Establishment, St. Andrews Road, Great Malvern, Worcestershire WR14 3PS, UK, 1976).

```
procedure detect;
  begin
    save-CPU-status;
    scan-keyboard;
    if seen then
      begin
        case character of
          M: pulsewidth modifier;
              (*regulate motor speed*)
          A: again;
              (*repeat operation*)
          ESC: abandone;
              (*stop operation*)
          F: fastspeed;
              (*accelerate motor*)
          s: slowspeed
              (*decelerate motor*)
          othercharacters: do-nothing
        end
      end;
            restore-CPU-status
  end
```

*Figure 8. On-line command recognizing routine.*

**LIQUID SCINTILLATION COUNTING**

*15–19 July 1985*

Another in the continuing series of Workshop Meetings on Liquid Scintillation Counting. The Workshop will be residential at Loughborough University of Technology, and will include lectures, practical work and discussion periods. The experimental work will be performed on a range of modern counters loaned by a number of companies (Amersham International, Beckmann RICC Ltd, Koch-Light Laboratories Ltd, Kontron Ltd, Laboratory Impex Ltd, LKB Instruments, Nuclear Enterprises Ltd, Packard Instruments Ltd, Philips Analytical, Pye Unicam Ltd and Rotheroe & Mitchel Ltd).

Papers include

A. Dyer: Instrumentation
P. Warwick: Standardization and efficiency determination
D. M. Taylor: Homogeneous sample preparation
G. Sutton: Automation and computing
P. Stanley: Safety.

The fee for the course will be £325 and includes accommodation in the University together with all meals. The number of places available is strictly limited and they will be allocated on a first-come first-served basis.

*The conference organizers are: Dr P. Warwick and Dr J. R. Thornback, Nuclear Chemistry Laboratories, Loughborough University of Technology, Loughborough, Leicestershire LE11 3TU, UK.*

